# The Necessity to Seal the Re-Entry Tears of Aortic Dissection After TEVAR: A Hemodynamic Indicator

**DOI:** 10.3389/fbioe.2022.831903

**Published:** 2022-03-31

**Authors:** Zhenfeng Li, Huanming Xu, Chlöe Harriet Armour, Yuze Guo, Jiang Xiong, Xiaoyun Xu, Duanduan Chen

**Affiliations:** ^1^ School of Life Science, Beijing Institute of Technology, Beijing, China; ^2^ Wenzhou Safety (Emergency) Institute of Tianjin University, Zhejiang, China; ^3^ Department of Chemical Engineering, Imperial College London, London, United Kingdom; ^4^ School of Biomedical Engineering, University of Sydney, Sydney, NSW, Australia; ^5^ Department of Vascular and Endovascular Surgery, Chinese PLA General Hospital, Beijing, China

**Keywords:** aortic dissection, re-entry tear, modified models, hemodynamic indicator, re-intervention or surgery

## Abstract

Thoracic endovascular aortic repair (TEVAR) is a common treatment for Stanford type B aortic dissection (TBAD). However, re-entry tears might be found distal to the stented region which transports blood between the true and false lumens. Sealing the re-entry tears, especially for the thoracic tears, could further reduce blood perfusion to the false lumen; however, it might also bring risks by re-intervention or surgery. Wise determination of the necessity to seal the re-entry tears is needed. In this study, patient-specific models of TBAD were reconstructed, and the modified models were established by virtually excluding the thoracic re-entries. Computational hemodynamics was investigated, and the variation of the functional index and first balance position (FBP) of the luminal pressure difference, due to the sealing of the re-entries, was reported. The results showed that the direction of the net flow through the unstented thoracic re-entries varied among cases. Excluding the re-entries with the net flow toward the false lumen may induce the FBP moving distally and the relative particle residence time increasing in the false lumen. This study preliminarily demonstrated that the hemodynamic status of the re-entry tears might serve as an indicator to the necessity of sealing. By quantifying the through-tear flow exchange and shift of FBP, one can predict the hemodynamic benefit by sealing the thoracic re-entries and thus wisely determine the necessity of further interventional management.

## Introduction

Aortic dissection is a life-threatening cardiovascular disease with high mortality (Hagan et al., 2000). It is usually treated by thoracic endovascular aortic repair (TEVAR) or open surgery (Dake et al., 1999; Qin et al., 2013; Nienaber et al., 2016). TEVAR is more commonly applied in treating Stanford type B aortic dissection (TBAD), even for uncomplicated TBAD patients, due to its favorite luminal remodeling and its lower mortality in mid- and long-term follow-ups (Qin et al., 2016). Recent studies investigated the risk factors that related to poor prognosis of TBAD after TEVAR (Trimarchi et al., 2013; Watanabe et al., 2014; Spinelli et al., 2018; Higashigaito et al., 2019) and reported that the thoracic re-entries played a vital role in the post-interventional prognosis (Tolenaar et al., 2013; Kotelis et al., 2016; Zhang et al., 2018). It was confirmed that the number of tears was associated with true and false lumen (TL and FL) development (Tolenaar et al., 2013; Kotelis et al., 2016), and it was suggested that the tears in the descending thoracic aorta should be repaired (Zhang et al., 2018). Moreover, an experimental study of TBAD based on an *ex vivo* platform indicated that the re-entry tears significantly affected the movement of the flap, which then influenced the flow pattern in the FL (Canchi et al., 2018). These studies raised the importance of the thoracic re-entries (Marui et al., 2007; Trimarchi et al., 2010; Tolenaar et al., 2013; Trimarchi et al., 2013; Evangelista et al., 2014; Song et al., 2014; Watanabe et al., 2014; Kotelis et al., 2016; Sato et al., 2017; Zhu et al., 2017). However, its hemodynamic role and the necessity to be occluded remained unanswered, for which quantitative hemodynamic analyses should be involved.

In recent years, hemodynamic computation has become a considerable way to investigate TBAD (Cheng et al., 2008; Chen et al., 2013b; Alimohammadi et al., 2014; Cheng et al., 2015; Sun and Chaichana, 2016; Xu et al., 2018). The flow pattern, the luminal flow exchange via the tears, the relative residence time that was related to thrombosis development, the luminal pressure interaction, etc. were quantitatively studied (Cheng et al., 2015; Xu et al., 2017; Xu et al., 2018; Pirola et al., 2019; Xu et al., 2020). Recently, we proposed a functional indicator to quantify the hemodynamic benefit by TEVAR and to predict post-TEVAR prognosis (Xu et al., 2020). It was based on the fact that the true and false luminal pressures interacted with each other and their difference related to the luminal development. By quantifying the shift of first balance position (FBP) of the luminal pressure difference curve, one can estimate the hemodynamic benefit by implanting the stent-graft (SG). In our previous study, it was confirmed that the shift of FBP was statistically related to the following luminal remodeling.

In this study, we aim to investigate the hemodynamic role of thoracic re-entry and to propose a method to evaluate the necessity of occlusion. The flow exchange via thoracic re-entry and its influence on FBP were investigated, and the relationships between these hemodynamic factors and following luminal development were analyzed.

## Materials and Methods

### Patients and Model Reconstruction

**FIGURE 1:**
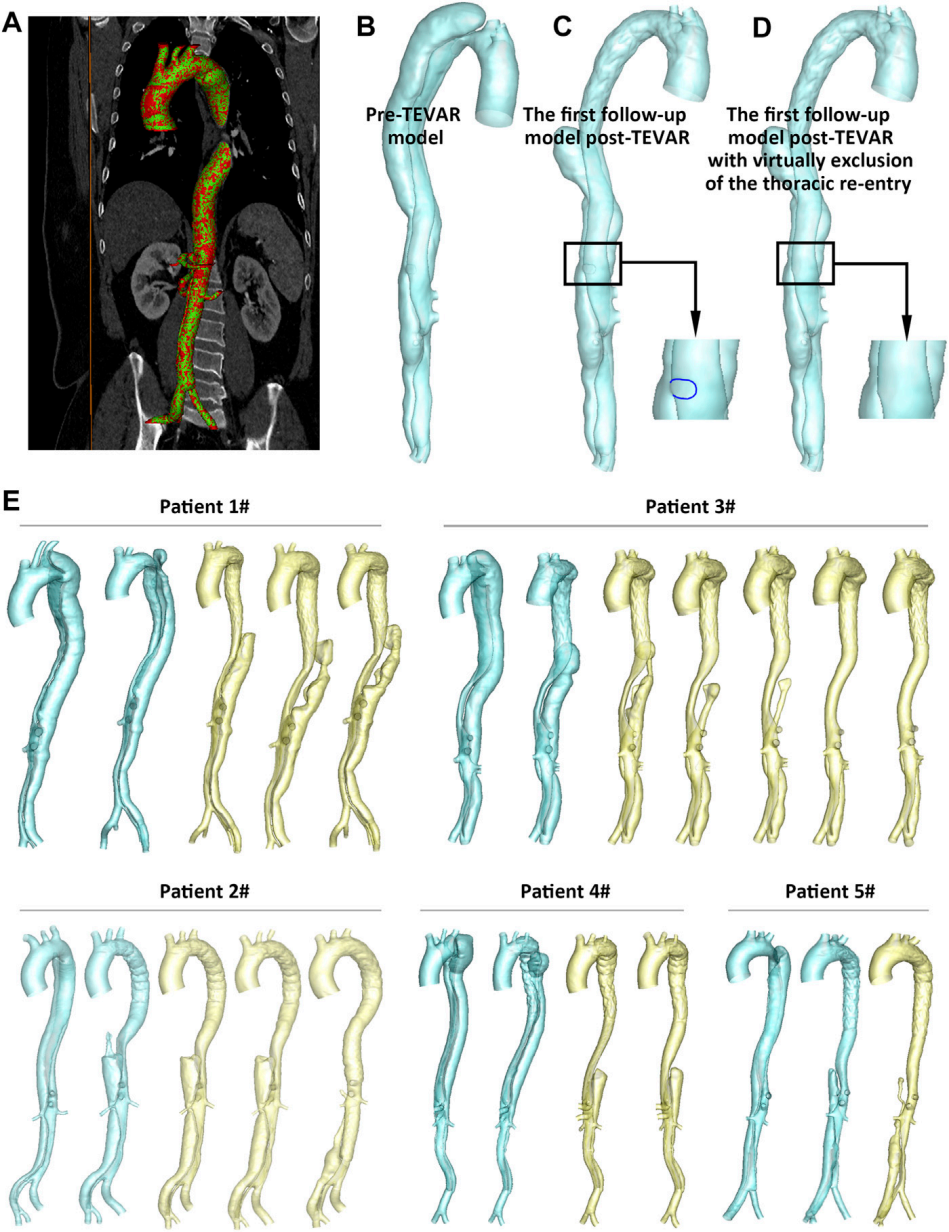
**(A)** Segmentation of the CTA image datasets and mapping back of the 3D reconstructed model to the original medical image. **(B–D)** As a representative, the pre- and post-TEVAR models and the artificially modified model of patient 2# were shown. **(E)** Geometric models of all of the cases with multiple image examinations were presented.

This study was approved by the Review Board of the Chinese PLA General Hospital (S201703601). Five patients with TBAD who underwent TEVAR and presented uncovered thoracic re-entries at the first post-TEVAR follow-up were included. The CTA data at initial presentation and multiple follow-ups after TEVAR were collected. Patient-specific models were established via image segmentation and 3D reconstruction, in Mimics 19.0 (Materialise, Belgium). As shown in Figure 1A, model surface smoothing was made, and the smoothed model was mapped back to the images to assess the accuracy of model establishment (Figure 1A).

To investigate the effects of the uncovered thoracic re-entries to the flow, the re-entry was artificially excluded to mimic the effect of sealing the tears. Thus, in the current study, patient-specific geometric models were generated based on the pre- and post-TEVAR image datasets, and five artificially modified geometric models were created based on the first-time follow-up model post-TEVAR. The pre-TEVAR and the first post-TEVAR (original and modified) geometric models were employed for hemodynamic computations, while the geometric models for the subsequent follow-ups were used for morphological analysis only, to quantify the luminal development and thus investigate its relationship to the hemodynamic conditions. All of the geometric models are displayed in Figure 1E.

By comparing the FL volume at the first and second post-TEVAR follow-ups (V_FL-1_ and V_FL-2_), FL remodeling status could be quantified by V_FL-2_-V_FL-1_; positive values indicated FL expansion, while negative values indicated FL reduction. By this means, the patient cases were categorized into two groups: FL expansion was found in patients 1# and 2# (group A), who experienced re-intervention to seal the thoracic re-entry post-TEVAR; the other patients (3#, 4#, and 5#) with stable FL remodeling were categorized as group B.

These models were imported into ICEM (Ansys 18.0, United States) for meshing with tetrahedral elements in the core region and prismatic cells (5 layers) in the boundary layer near the aortic wall. The elements in these models varied from 3 to 4.5 million. The meshing sensitivity test was conducted in the previous study, indicating the number of elements used in this study was adequate (Xu et al., 2017).

### Numerical Simulation and Boundary Conditions

According to the previous studies, blood was treated as Newtonian fluid, with the dynamic viscosity and density of blood as 0.00365 Pa-s and 1,060 kg/m^3^, respectively. The inlet of each model was assigned as velocity inlet with a flat profile, and the flow waveform was assigned based on a previous study (Dillon-Murphy et al., 2016). The 3-element Windkessel model, which could capture the distal outlet resistance and compliances of the vessel, was assigned at each outlet with the relevant parameters taken from the same study (Dillon-Murphy et al., 2016). The vessel wall was regarded as no slip and rigid, owing to the low distensibility of aorta/arteries in patients with aortic dissection (Chen et al., 2013a; Alimohammadi et al., 2014; Cheng et al., 2014; Cheng et al., 2015). The time step was set to 0.005 s, and the time-step sensitivity test was conducted in one previous study as well (Menichini et al., 2018).

All flow simulations were run on CFX 18.0 (ANSYS, United States) to solve the transport equations—Navier–Stokes equations, together with the continuity equation of incompressible and Newtonian fluid (Eqs. 1, 2), where *u* stands for velocity, ρ stands for density, *μ* represents dynamic viscosity, and *P* denotes pressure. Simulations were carried out for four cardiac cycles for each model to achieve periodic solutions, and the results of the last cycle were selected for post-processing. The convergence of the solution was controlled by specifying a max root-mean-square of 10^−6^.

∇⋅(u)=0 (1)

$$\rho \left(\frac{\partial u}{\partial t}\right)+\rho \left(u\cdot \nabla \right)u=-\nabla P+\mu \nabla^{2}u$$ (2)

## Results

### Morphological Analysis

**FIGURE 2:**
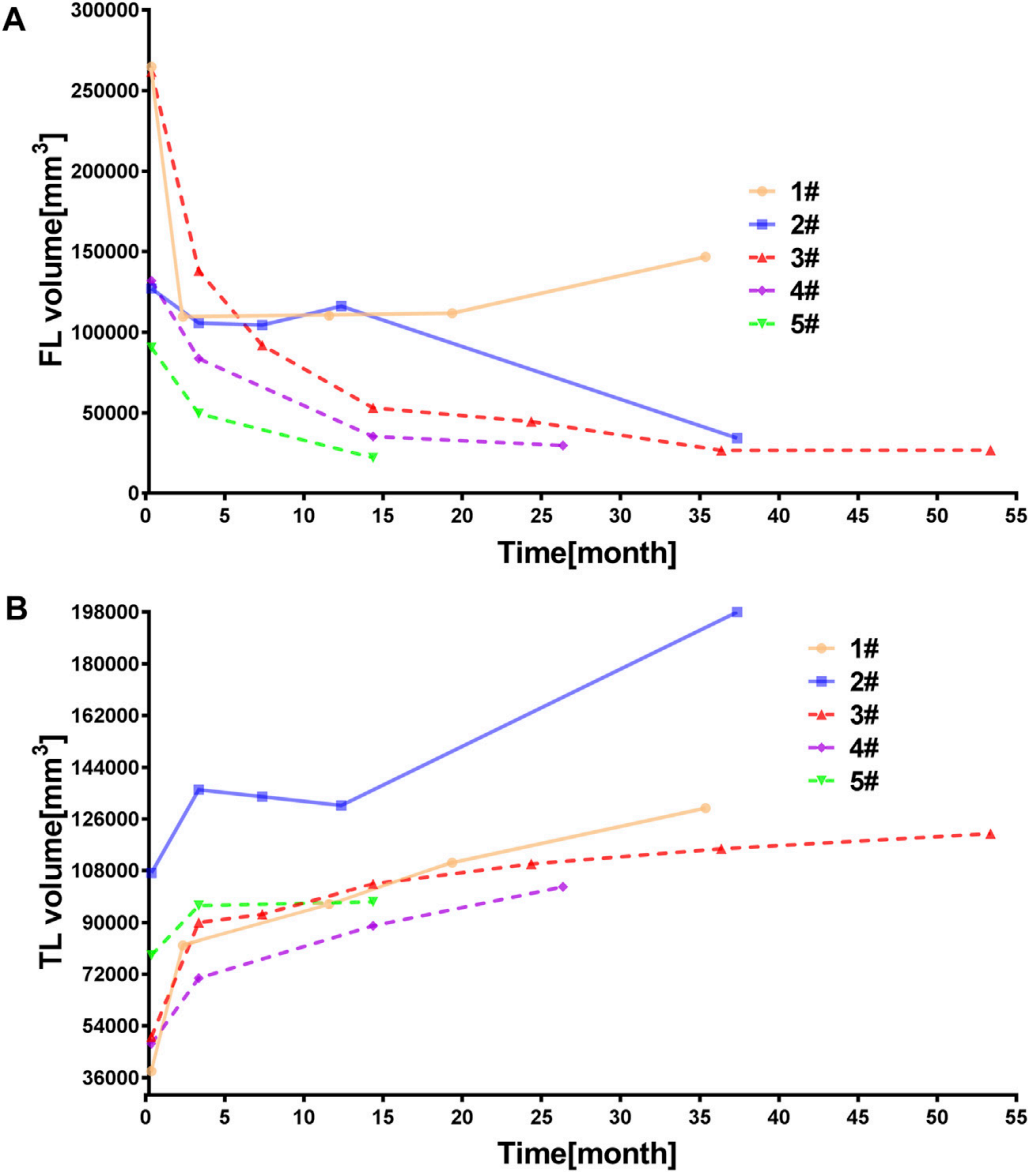
**(A–B)** Variation of the false and true lumen volume changes during follow-up.

Several morphological parameters were calculated in this study, including the number of tears, tear size, and the luminal volumes. Figure 2 indicates the volume of TL and FL and their variation during the follow-up. By comparing the FL volume at the second and first follow-up post-TEVAR (V_FL-2_-V_FL-1_), patients 1# and 2# presented the potential for further FL enlargement (Figure 2A) and were thus categorized to group A. For those patients in group B, the FL volume decreased during the whole period of follow-up. The variations of TL volume showed some difference as only TL volume of patient 2# decreased trivially. The TL volume increased for the other case including patient 1#, as displayed in Figure 2B.

Table 1 listed the detailed information of the tears including primary tear before TEVAR and the re-entry tears at the first-time follow-up after TEVAR. The case with the largest primary tear size was patient 4# with 407 mm^2^, followed by patient 3# with an area of 386.0 mm^2^. Both of them belonged to group-B. Furthermore, patient 3# was associated with the largest area of total re-entry tears, and the thoracic re-entry of which was closed in the next few months after the first time follow-up post-TEVAR, as shown in our previous study (Xu et al., 2017). There were 9 re-entry tears in patient 2#, while the area of tears was relatively low (126.3 mm^2^) due to the small size of each tear. The smallest thoracic re-entry appeared in patient 4# with a size of merely 7 mm^2^.

**TABLE 1:** Tear information of patients included in this study.

Group	Patient	Number of tears^a^	Location of the tears^b^ (mm)			Area of primary tear^c^ (mm^2^)	Area of total tears^d^ (mm^2^)	Area of the thoracic re-entry tear^d^ (mm^2^)
			Tear no	Linear distance	Curve distance			
A	1#	3	1	23.7	24.7	160.0	169.8	62.6
			2	194.8	252.2			
			3	345.4	428.7			
	2#	8	1	32.7	33.7	156.3	126.3	23.0
			2	185.0	234.2			
			3	224.6	277.3			
			4	282.8	340.7			
			5	323.2	380.2			
			6	340.6	400.7			
			7	371.4	436.9			
				8	418.1				509.8
B	3#	3	1	54.9	57.7	386.0	221.5	70.7
			2	265.4	358.7			
			3	362.4	464.6			
	4#	6	1	23.2	24.4	407. 2	50.2	7.0
			2	31.6	42.2			
			3	183.4	247.8			
			4	212.8	280.0			
			5	261.3	278.0			
			6	360.4	383.4			
	5#	3	1	23.6	23.9	187.6	114.2	42.1
				2	289.0				328.5
				3	342.4				380.5

a Tears along the aorta were counted and measured, while those in the iliac arteries were not included.

b Centerline of the true lumen was extracted for each geometric model. The highest position which was presented in the aortic arch region was assigned as the reference point for each model, and the straight line distance and the curve distance along the centerline between this reference point to the centroid of each tear were measured, regarded as the location of the tears.

c Indicated the data measured in the geometric models before TEVAR.

d Indicated the data measured in the geometric models at the first-time follow-up examination after TEVAR.

### Luminal Pressure Difference and the First Balance Point

**FIGURE 3:**
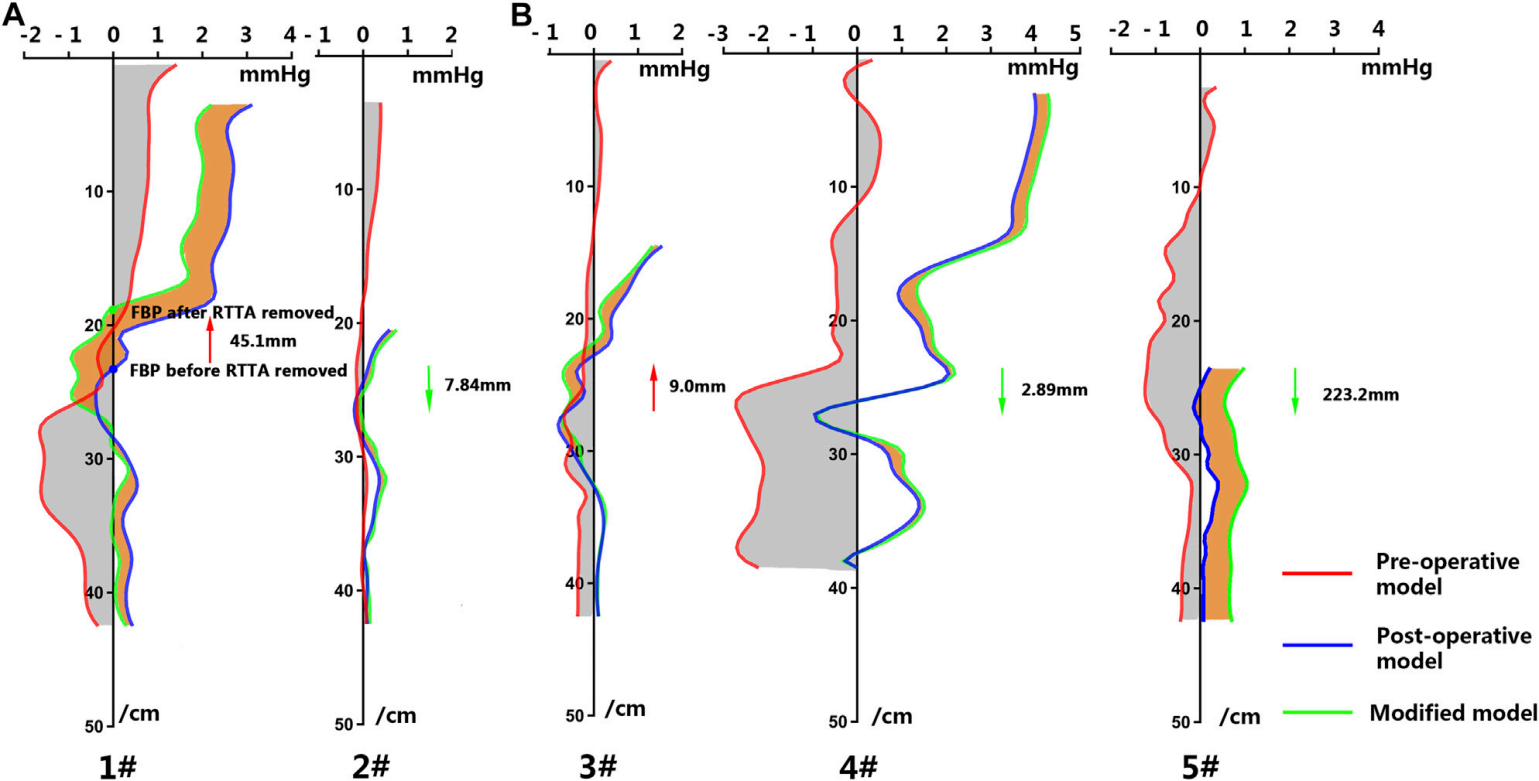
Luminal pressure difference (LPD) along the central line of the true lumen for the patients in group A **(A)** and group B **(B)**.

The luminal pressure difference (LPD) from the proximal dissection to the iliac bifurcation was calculated over a cardiac cycle via the same method as our previous study (Xu et al., 2020). A series of cross-slices that were perpendicular to the central line of TL were created, and the averaged pressure of these slices over the cardiac cycle was calculated. The LPD in each slice equaled to P_TL_-P_FL_, and the LPD curve along the central line of TL in all patients is shown in Figure 3. Moreover, the FBP, which indicated the first position of equality pressure in TL and FL (PD = 0), was investigated in this study. The distance between the root of the proximal subclavian artery and the iliac bifurcation along the central line of TL was normalized to 424 mm for all cases, and the movement distance of FBP before TEVAR and the first follow-up after TEVAR was measured in all patients.

According to the conclusion of our previous study, the location of the FBP post-TEVAR was related to the prognosis of long-term follow-up. The distal shifts of FBP of these cases in group A were 29.2 and 60.23 mm, while in group B, the shifts were 95.8, 153.9, and 151.73 mm. If the thoracic re-entry was excluded, the FBP would move up proximally for patients 1# (45.1 mm) and 3# (9 mm), as the red arrow indicated in Figure 3. As for patients 2# and 4#, the FBP would move down with a distance of 7.84 and 2.89 mm, respectively. For patient 5#, if the thoracic re-entry was excluded, there was no balance point, which meant the FBP moved down beyond the studied aortic region, and the distance between the FBP and the iliac bifurcation was 174.1 mm. These results indicated that the direction of the shift of FBP varied among different cases on the condition of the exclusion of the thoracic re-entry. In other words, the thoracic re-entry affected the location of FBP and may induce different prognosis for TBAD post-TEVAR.

### Flow Exchange Between True Lumen and False Lumen

**FIGURE 4:**
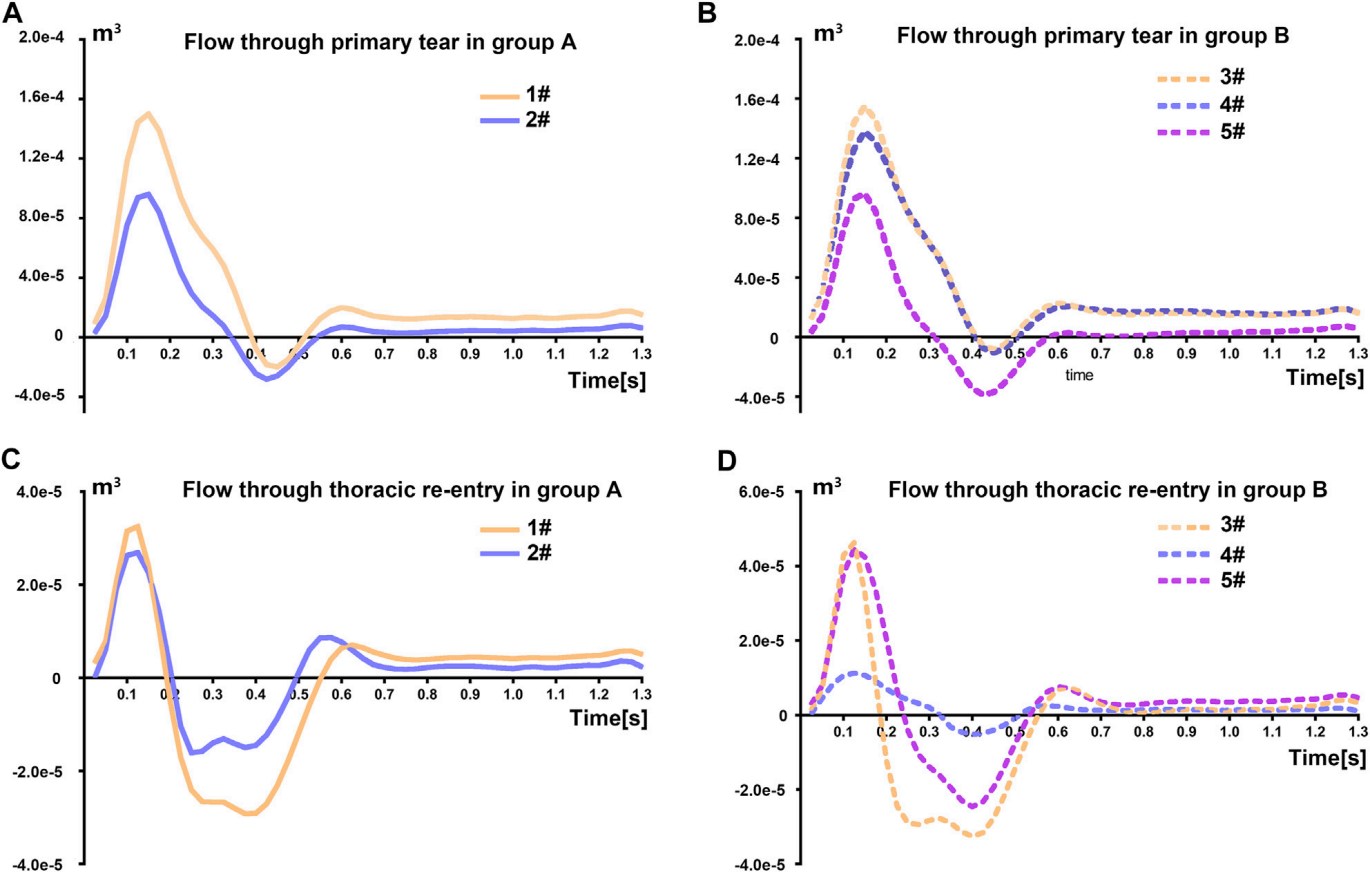
**(A–B)** Time-variant flow volume entering the false lumen via the primary tear of the patients. **(C–D)** The time-variant flow volume entering the false lumen via the thoracic re-entry tear of the patients.

The flow entering the FL via the primary tear was regarded as a key factor that may relate to the prognosis after TEVAR. Figure 4A and Figure 4B showed the flow rate variations through the primary tear for both groups. The results indicated that the primary tear acted as the inlet to the FL for almost the entire cardiac cycle, especially for patients 3# and 4#, in which the area of the primary tear was relatively large. The flow split ratio, which indicated the percentage of flow rate through the specific tear of surface over the total flow of inlet, through the primary tear for patients 1# and 2# in group A was 41.80 and 16.29%, respectively, while that for patients 3#, 4#, and 5# of group B was 46.81, 10.95, and 44.75%, respectively.

The flow entering FL over a cardiac cycle through the thoracic re-entry and the total flow exchange in the descending aorta were calculated for all the cases after TEVAR, as given in Table 2. Negative values indicated that the re-entry tears contributed to negative transportation of the blood toward the FL. Figure 4C and Figure 4D display the flow rate exchange over a cardiac cycle. For the thoracic re-entry in group A, the tear mainly acted as the inlet to the FL during early systole, while it acted as the outlet of FL in the late systole and during the entire diastole. Moreover, the areas of the tear affected the flow exchange. In patient 4#, the area of thoracic re-entry was merely 7.0 mm^2^ and the corresponding flow exchange was small, while for patients 1# and 3#, the thoracic re-entry areas were 62.6 and 70.7 mm^2^, respectively, which induced a relatively larger flow exchange.

**TABLE 2:** Flow exchange through the re-entry tears and the false lumen after TEVAR.^a^

Patient	Flow exchange through thoracic re-entry^b^ [%]	Total flow exchange through tears [%]	Total flow exchange after exclusion of the thoracic re-entry tear [%]
1#	-4.40	11.20	7.84
2#	2.29	6.23	6.18
3#	-2.91	15.59	5.09
4#	2.69	13.56	12.15
5#	2.29	6.89	4.34

a Flow exchange was presented as the through-tear flow ratio to the inflow at the inlet of the ascending aorta.

b In this column, positive values indicated blood volume transported from true lumen to the false lumen, while negative values indicated reversed flow from the false lumen to the true lumen.

Table 2 quantify the flow exchange through the re-entry tears and the total flow that passes the FL after TEVAR. The results indicated that the thoracic re-entry contributed to positive transportation of the blood toward the FL for patients 2#, 4#, and 5#, with the flow splitting to FL by 2.29%, 2.69%, and 2.29%, respectively, while the thoracic re-entry tear contributed to negative transportation of the blood toward the FL for patients 1# and 3#, with a flow split of 4.40% and 2.91%, respectively. The amount of the flow varied among the cases based on their area of tears and aortic geometry. The total flow splitting to the FL through all re-entries in the descending aorta after TEVAR varied from 6.23% to 15.59% in all patients. The FL flow splitting ratio declined in varying degrees when the thoracic re-entry tears were blocked.

### Relative Residence Time

**FIGURE 5:**
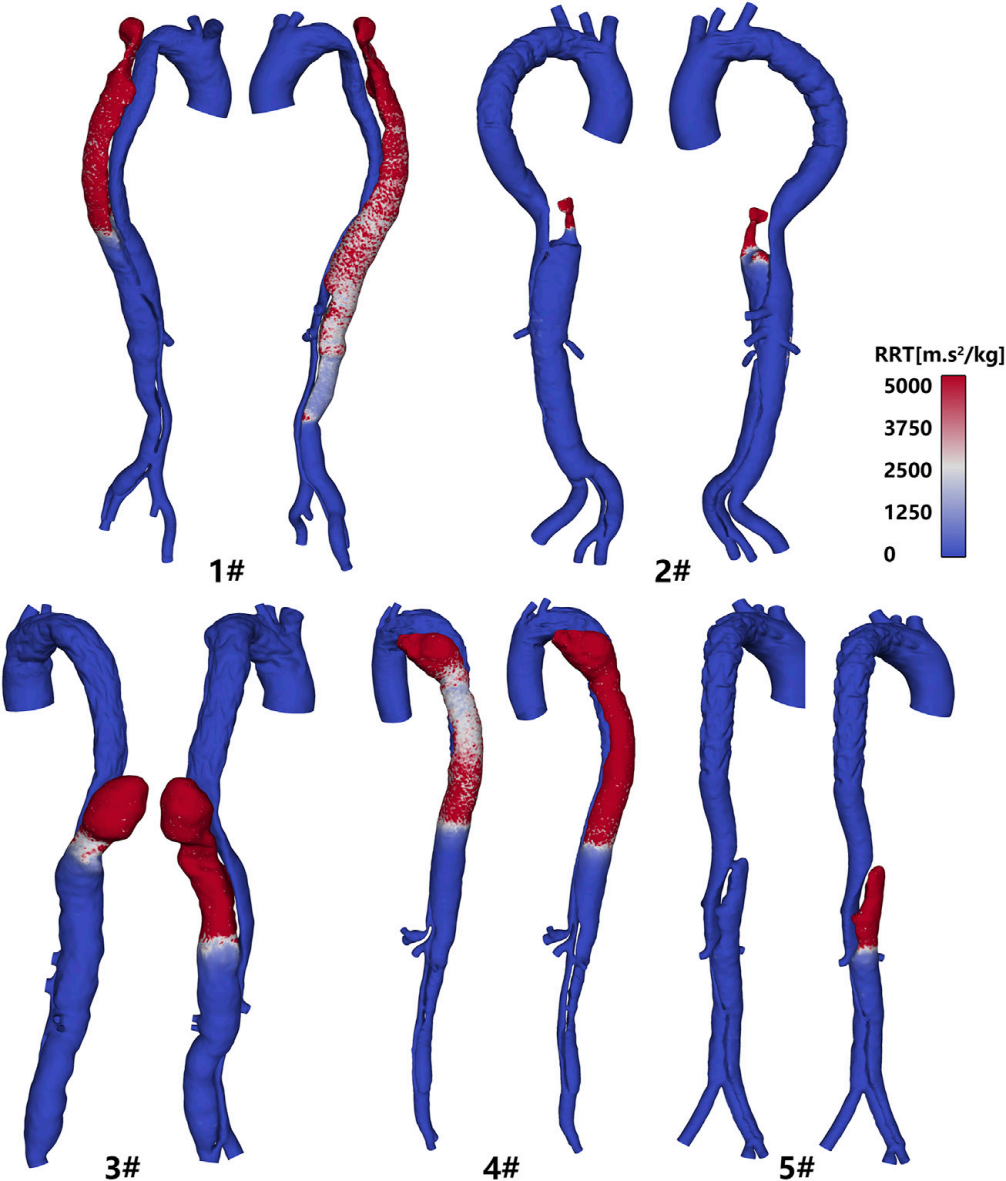
Distribution of the relative residence time in each computational model. In each case, the post-TEVAR model is on the left, and the modified model is on the right.

Relative residence time (RRT) is a key hemodynamic parameter that could disclose the potential thrombosis in FL to some degree. The values of RRT before and after the thoracic re-entry was sealed were compared in this study. It could be observed in Figure 5 that the RRT was higher at the proximal tip of the FL for those models, when the thoracic re-entry was excluded. The thrombosis process would be sped up comparing to the original aortic geometry without re-entry exclusion (Armour et al., 2020). As shown in Figure 5, the region where RRT was lower than 5,000 increased significantly when the thoracic re-entry was removed. These cases presented a longer distance between the location of re-entry tear and the proximal tip of FL. For patient 2#, the second re-entry tear was located close to the proximal tip of FL; thus, the difference of RRT distribution was trivial before and after the thoracic re-entry was removed.

## Discussion

Re-entry tears played an important role in the prognosis of post-TEVAR patients. The flow would strike the vessel wall in the FL through thoracic re-entry since the blood flow entered into FL around the systolic peak, as shown in Figure 4C, leading to higher risk of FL expansion. However, whether sealing the thoracic re-entries is beneficial to all patients remains controversial. The flow exchange through thoracic re-entry might induce further FL expansion, while sealing these tears by SG might cause additional risk during intervention (Zhang et al., 2018). The necessity to exclude thoracic re-entry should be further clarified. Previous studies showed that TBAD patients benefited from the absence of distal re-entry tears (Zhu et al., 2017) and the number of re-entry tears closely related to aortic growth (Tolenaar et al., 2013). In our study, we aim to further reveal the hemodynamic significance of the thoracic re-entry, investigate its relation to the prognosis after TEVAR, and propose a method to evaluate the necessity to interfere thoracic re-entry from the hemodynamic perspective.

**FIGURE 6:**
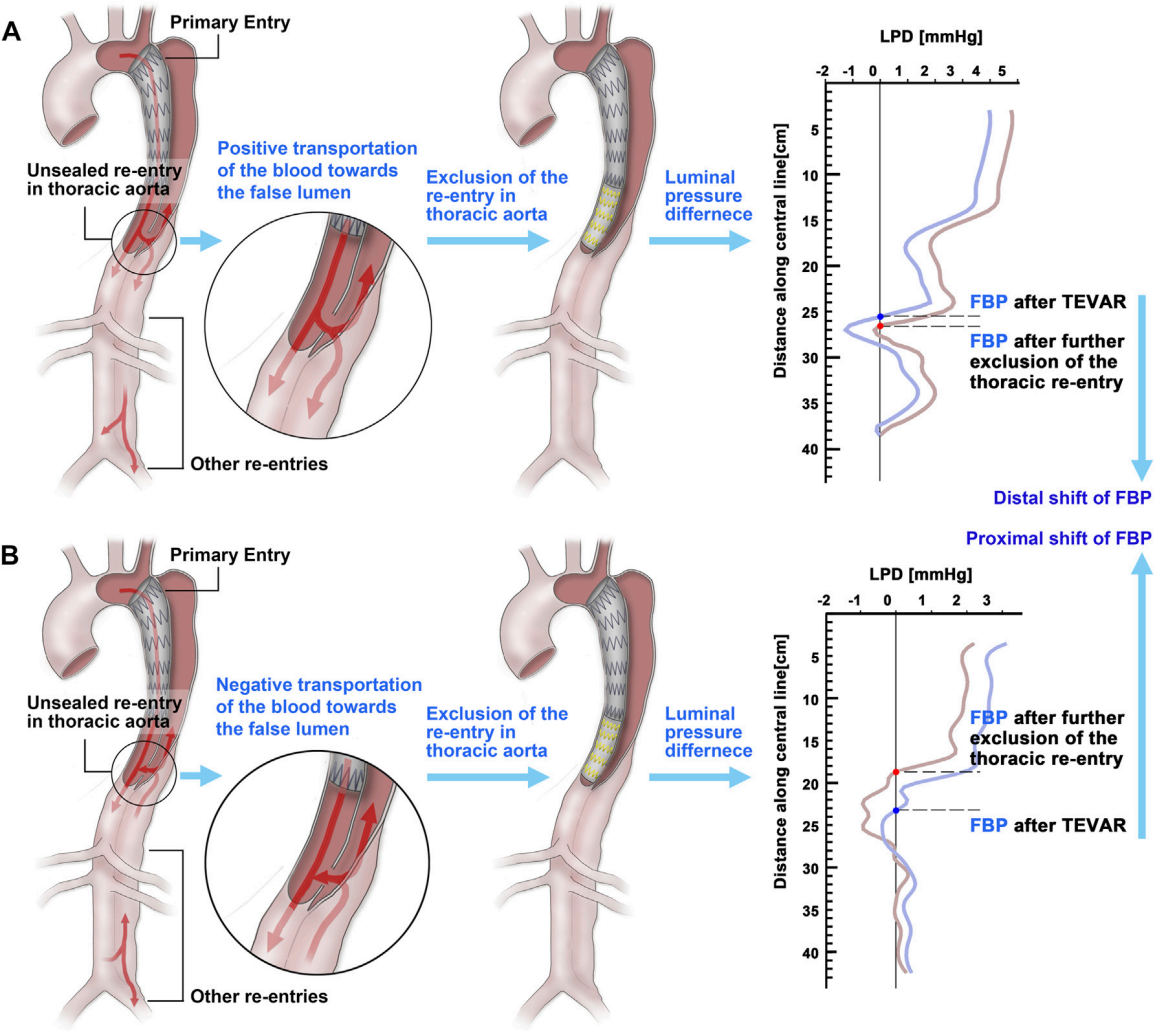
Shift direction of first balance position (FBP) of the luminal pressure difference. **(A)** The FBP moves to the distal region if excluding the thoracic re-entry when it contributes to positive transportation of the blood toward the false lumen. **(B)** The FBP moves to the proximal region if excluding the thoracic re-entry when it contributes to negative transportation of the blood toward the false lumen.

As a preliminary study, five TBAD cases were studied. Two of them underwent the second intervention post-TEVAR to exclude the thoracic re-entries due to the enlargement of FL. At first, the distal shift of FBP after TEVAR was calculated and compared between group A (enlarged FL) and group B (stable or vanished FL). The shift of FBP in group A was smaller than that in group B (44.72 ± 15.52 mm vs 133.8 ± 32.9 mm), which was consistent with the results of our previous study (Xu et al., 2020), and indicated that the hemodynamic benefit by TEVAR in group B was much greater than that in group A. Then, the influence of thoracic re-entry exclusion on FBP was investigated. As illustrated by Figure 6, it was found that the direction of the shift of FBP by sealing the thoracic re-entry was dependent on the hemodynamic role of these tears. If the thoracic re-entry contributed to positive transportation of the blood toward the FL, the exclusion of thoracic re-entry would induce distal shift of FBP (Figure 6A), while if the thoracic re-entry contributed to negative transportation of the blood toward the FL, sealing it might induce proximal movement of FBP (Figure 6B). As demonstrated by our previous study (Xu et al., 2020), distal positioned FBP indicated positive effect for luminal development, while the proximal shift of FBP implicated that sealing this type of thoracic re-entry might not bring positive contributions to luminal remodeling. For instance, there are three re-entry tears in patient 1#. The split flow ratio entering into the FL through the second re-entry tears was 11.2%, while it decreased to 7.84% when the thoracic re-entry was excluded. The reduction of flow entering into the FL may reduce the impact of flow on the wall and contribute to positive thrombosis formation in the FL. Thus, the hemodynamic role of thoracic re-entry may be an indicator to evaluate the necessity to seal the re-entry of post-TEVAR TBAD.

In the current study, sealing of the thoracic tear was applied to patients 1# and 2# via re-intervention. Based on the proposed hemodynamic indicator, occlusion of the re-entry in patient 1# might not be beneficial; however, that for patient 2# might have potential to promote positive luminal remodeling. During the long-term follow-ups of these two patients, it could be revealed that continuous expanding of the FL was presented in patient 1#, while significant FL reduction was shown in patient 2#. Although the cases involved in the current study were limited, the results showed the potential of the hemodynamic indicator to better determine the necessity of tear exclusion.

The thrombosis process in the FL affects the prognosis post-TEVAR (Song et al., 2014; Menichini et al., 2018). It was found the thoracic re-entry influenced the fluid environment of FL and thus affected thrombosis development. As shown in Figure 5, the cases with thoracic re-entry exclusion exhibited larger RRT, indicating the blood flow was stagnant at the top of FL, and surface thrombosis would be generated. This was consistent with the previous study which confirmed that thrombosis was more likely to be established in the FL in patients without thoracic re-entry (Armour et al., 2020).

The re-entry tear is an important influential factor to the pressure environment of TBAD; however, other factors also exist. For instance, the FL branches, which were reported to be related to complications (Ge et al., 2017; Liu et al., 2018), may also affect the pressure distribution. However, in the current study, similar result was not found as there were no FL branches involved in patients 1# and 2#, while the dissection of patients 3# and 4# was associated with branches, which indicate the involved branches played an insignificant role for the cases included in this study. A hemodynamic study is a significant supplement to the clinic statistic study, which could provide functional parameters related to prognosis. Previous studies concluded that the thoracic re-entry contributed to positive transportation of the blood toward the FL (Zhang et al., 2018). However, the results in our study showed that the tear might also contribute to negative transportation of the blood toward the FL for some patients.

As a preliminary study, there are a few limitations in this study such as the rigid wall assumption and small number of cases. Due to the complex geometry and the lack of the actual material properties, the existing fluid–structure interaction studies on TBAD often generated the aortic/dissection wall with arbitrary thickness and assumed the mechanical properties of the wall/flap as linear elastic. More accurate simulations are highly dependent on accurate model establishment and material property measurements, which are currently being carried out in our laboratory. Since the TBAD patients who presented thoracic re-entry post-TEVAR and with routinely examined follow-up images were limited in our center, only five cases were recruited in the current study. Continuous data collection is carried out in our center, and more convincing conclusion could be drawn in the future.

## Conclusion

The current study investigated the hemodynamic significance of the unstented thoracic re-entry of TBAD. The results indicated that i) due to the various morphological condition of each patient, the thoracic re-entry might contribute to positive or negative transportation of the blood toward the false lumen; ii) sealing the thoracic re-entry tears that presented positive flow contributions to the false lumen would induce distally shifting of the first balance position of the luminal pressure difference curve, and *vice versa*. Comparing to the long-term follow-up results of the luminal remodeling, this preliminary study implicated that a hemodynamic role might be a more effective indicator to determine the necessity to seal the thoracic re-entries. This might contribute to the wise decision-making of re-intervention or surgery after TEVAR.

## Data Availability

The original contributions presented in the study are included in the article/Supplementary Material, further inquiries can be directed to the corresponding authors.
